# Supplementary material for: OsMADS17 simultaneously increases grain number and grain weight in rice
Source: Nat Commun. 2023 May 29;14:3098. doi: 10.1038/s41467-023-38726-9 (PMC10227085; doi:10.1038/s41467-023-38726-9)
Supplement: Supplementary file 1 — Supplementary Information [file 41467_2023_38726_MOESM1_ESM.pdf]

***OsMADS17* simultaneously increases grain number and grain weight in rice**

Li *et al*.

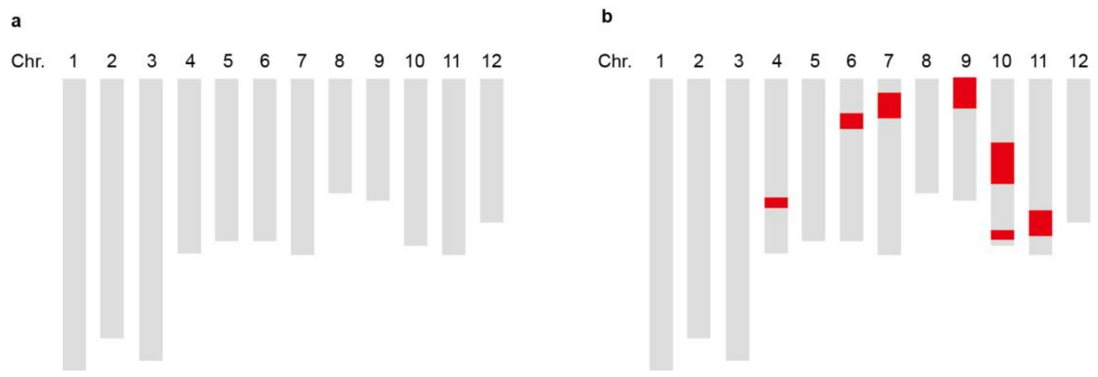

Supplementary Figure 1. Presentation of graphical genotypes of C418 (a) and 8IL73 (b). The gray regions represent C418 genetic background, and the red regions indicate the chromosomal introgression fragments from Dongxiang common wild rice (DXCWR).

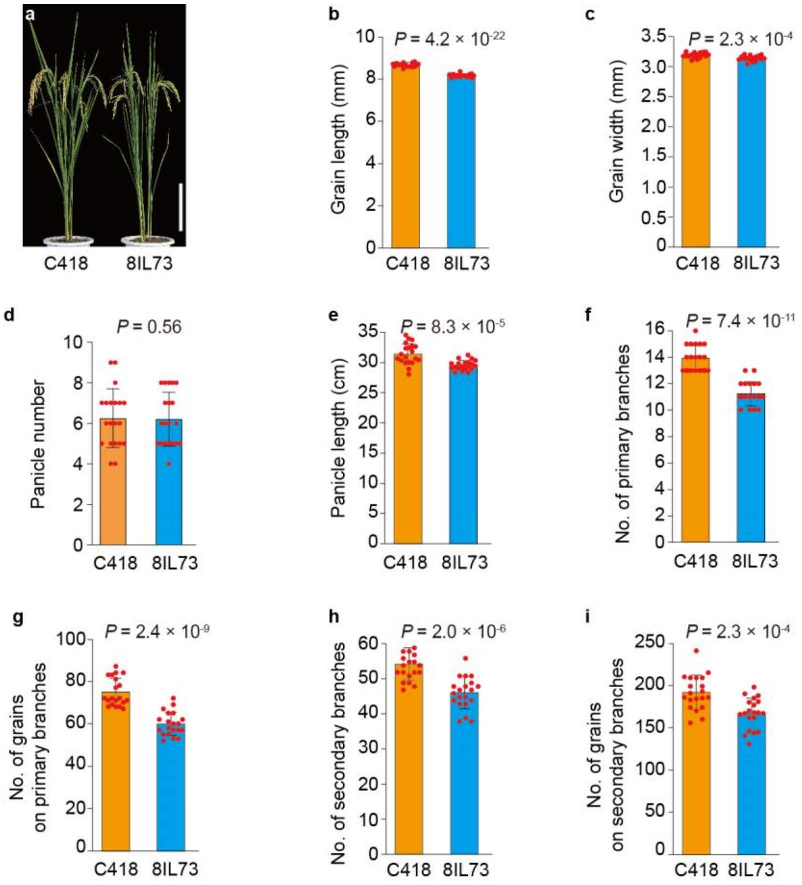

Supplementary Figure 2. Comparison of agronomic traits between C418 and 8IL73 plants. **a** The morphology of C418 and 8IL73 plants. Scale bar, 25 cm. **b**-**i** Comparison of grain length (**b**), grain width (**c**), panicle number (**d**), panicle length (**e**), number of primary branches (**f**), grain number on primary branches (**g**), number of secondary branches (**h**), and grain number on secondary branches (**i**) between C418 and 8IL73. Data are means ± s.d. (n = 20 plants), comparisons are made by two-tailed Student's t-test. Source data underlying Supplementary Fig. 2b-i are provided as a Source Data file.

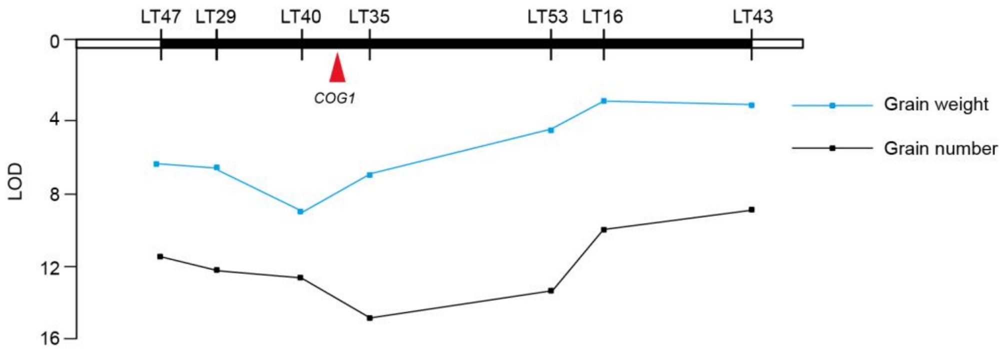

Supplementary Figure 3. Quantitative trait locus (QTL) analysis. A QTL for co-regulating grain number and 1,000-grain weight was detected by single-point analysis using 203 F_2 individuals and seven pairs of primers. LOD (likelihood of odd) was performed by the software Map Manager QTXb20. Source data are provided as a Source Data file.

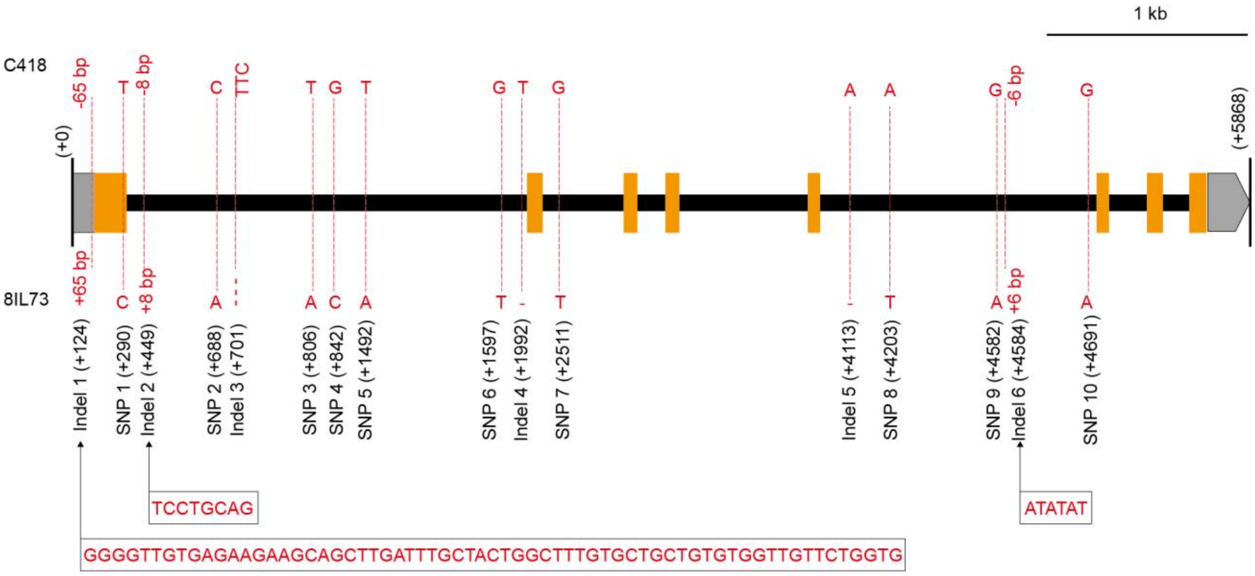

Supplementary Figure 4. Sequence comparison of the *OsMADS17* gene between C418 and 8IL73. The *OsMADS17* gene comprises eight exons and seven introns. The black bold lines represent introns, the orange boxes represent coding sequences (CDS), and the gray boxes represent 5′ UTR and 3′ UTR. Sequence variations between C418 and 8IL73 are marked by red letters. Position marked in the bracket is referenced to the *OsMADS17* gene according to the UGA (previously MSU) Rice Genome Annotation Project Database (http://rice.uga.edu/).

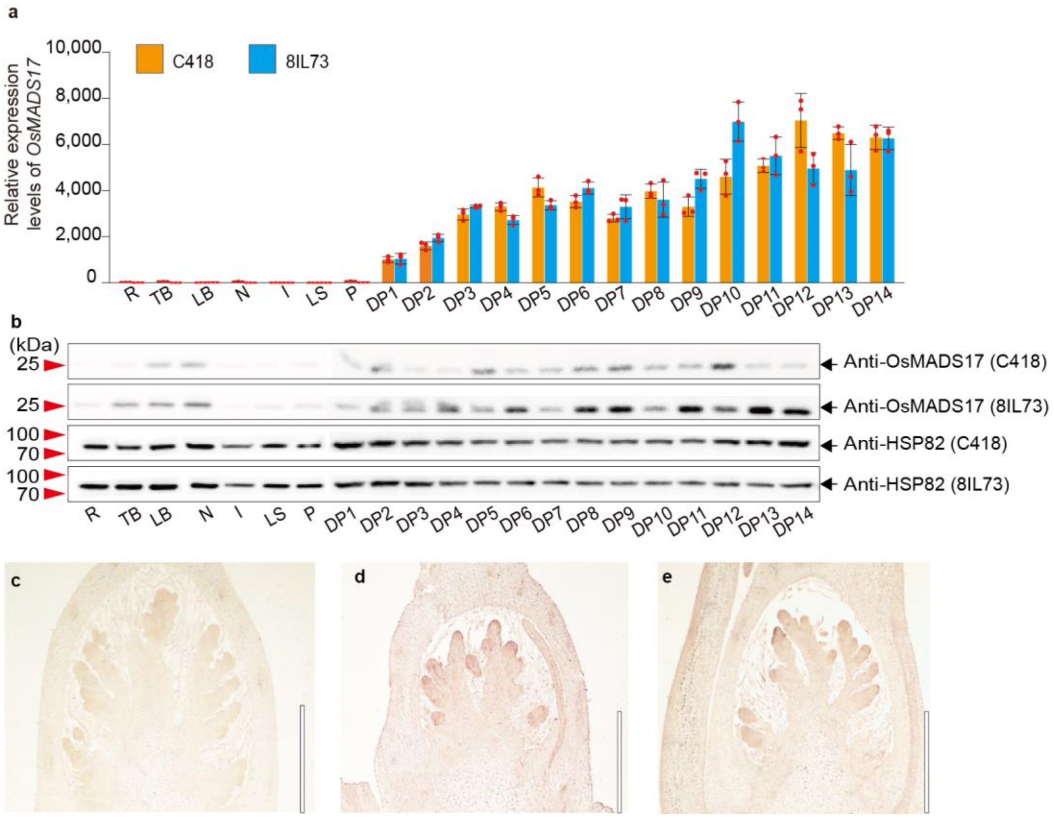

Supplementary Figure 5. Tissue- and development-specific expression patterns of *OsMADS17*. a Relative expression of *OsMADS17* in C418 and 8IL73. R, root; TB, tiller base; LB, leaf blade; N, node; I, internode; LS, leaf sheath; P, pulvinus; DP1–DP14, developing panicles; 0–0.2 cm (DP1); 0.2–0.4 cm (DP2); 0.4–0.6 cm (DP3); 0.6–0.8 cm (DP4); 0.8–1.0 cm (DP5); 1.0–1.5 cm (DP6); 1.5–2.0 cm (DP7); 2.0–2.5 cm (DP8); 2.5–3.0 cm (DP9); 3.0–4.0 cm (DP10); 4.0–5.0 cm (DP11); 5.0–10 cm (DP12); 10–15 cm (DP13); 15–20 cm (DP14). Data are means ± s.d (n = three replicates). **b** Same as (**a**) for western blot analysis for OsMADS17. HSP82 was used as the loading control (n = three replicates). **c**-**e** Demonstration of *OsMADS17* expression patterns by RNA *in-situ* hybridization. Sense probe as negative control in the 8IL73 young panicle (**c**), RNA *in-situ* hybridization of *OsMADS17* in the young panicle of C418 (**d**) and 8Il73 (**e**) (n = three replicates). Scale bars, 500 μm. Source data underlying Supplementary Fig. 5a, b are provided as a Source Data file.

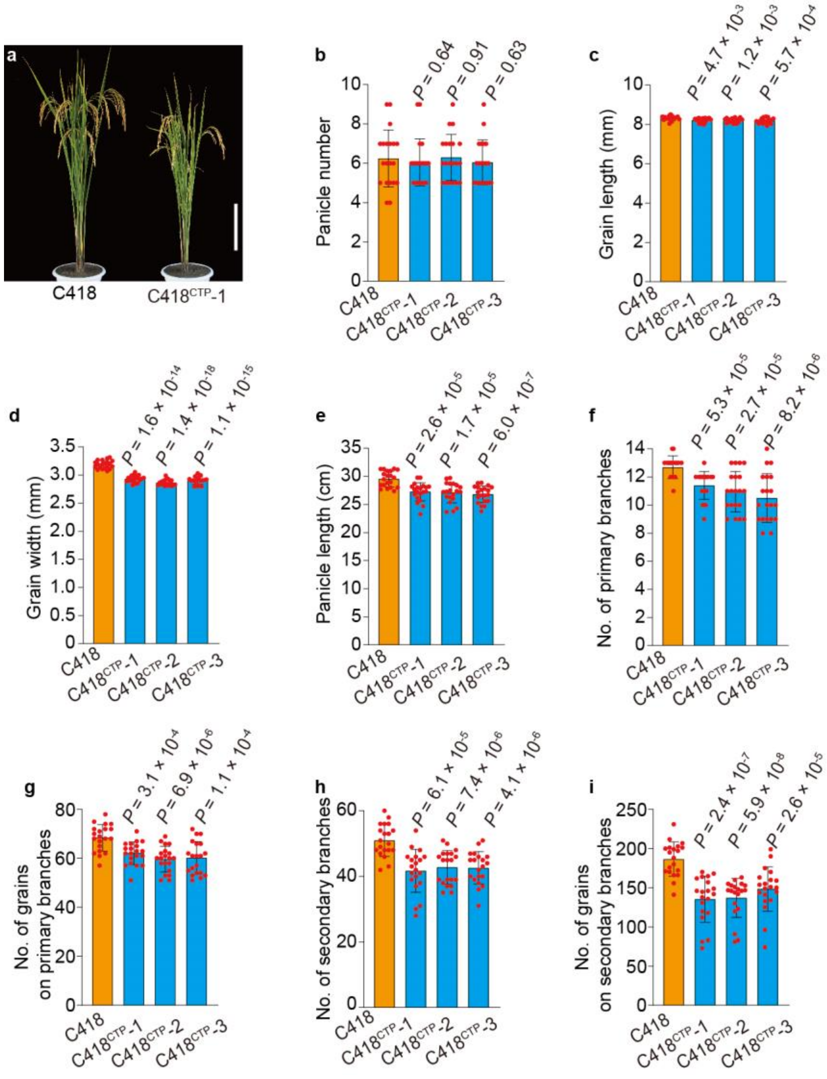

Supplementary Figure 6. Comparison of agronomic traits between C418 and C418^{CTP} plants. **a** Plant morphology of C418 and C418^{CTP} plants. Scale bar, 25 cm. **b-i** Comparison of panicle number (**b**), grain length (**c**), grain width (**d**), panicle length (**e**), number of primary branches (**f**), grain number on primary branches (**g**), number of secondary branches (**h**), and grain number on secondary branches (**i**) between C418 and C418^{CTP} plants. Data are means ± s.d. (n = 20 plants), comparisons are made by two-tailed Student's t-test. Source data underlying Supplementary Fig. 6b-i are provided as a Source Data file.

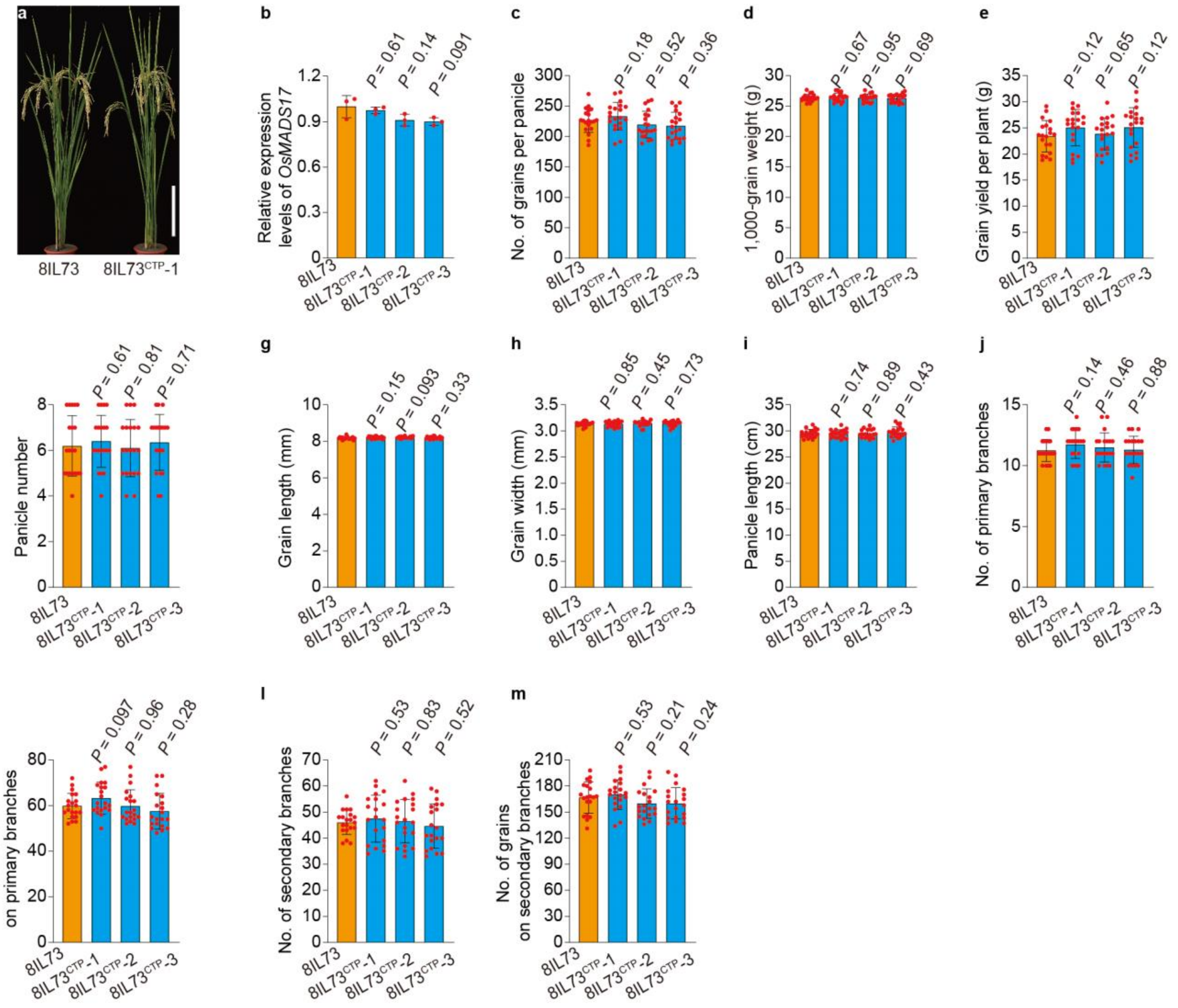

Supplementary Figure 7. The *OsMADS17* gene from C418 showed no obvious effects on grain yield regulation. a Plant morphology of 8IL73 and 8IL73^{CTP} plants. Scale bar, 25 cm. **b** Relative expression levels of *OsMADS17* in 8IL73 and 8IL73^{CTP} plants (*n* = three replicates). **c**-**m** Comparison of grain number (**c**), 1,000-grain weight (**d**), grain yield per plant (**e**), panicle number (**f**), grain length (**g**), grain width (**h**), panicle length (**i**), number of primary branches (**j**), grain number on primary branches (**k**), number of secondary branches (**l**), and grain number on secondary branches (**m**) between 8IL73 and 8IL73^{CTP} plants. Data are means ± s.d. (*n* = 20 plants), comparisons are made by two-tailed Student's *t*-test. Source data underlying Supplementary Fig. 7b-m are provided as a Source Data file.

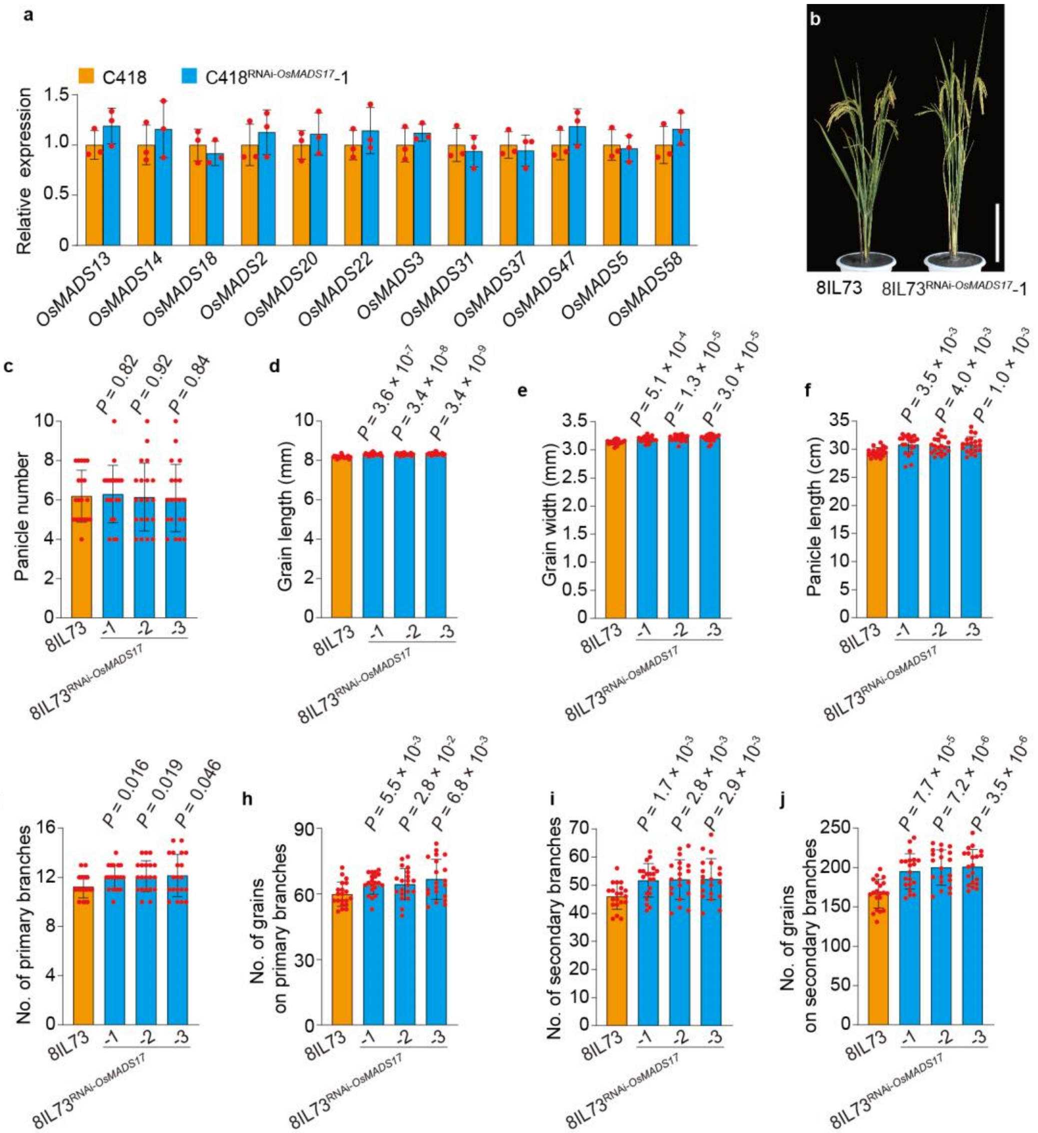

Supplementary Figure 8. Comparison of agronomic traits between 8IL73 and 8IL73$^{RNAi\text{-}OsMADS17}$ plants. a Identification of specificity for *OsMADS17* specific RNA interference (RNAi) vector by testing the expression levels of 12 MADS family genes in transgenic and the control plants (*n* = three replicates). **b** Plant morphology of 8IL73 and 8IL73$^{RNAi\text{-}OsMADS17}$ plants. Scale bar, 25 cm. **c**-**j** Comparison of panicle number (**c**), grain length (**d**), grain width (**e**), panicle length (**f**), number of primary branches (**g**), grain number on primary branches (**h**), number of secondary branches (**i**), and grain number on secondary branches (**j**) between 8IL73 and 8IL73$^{RNAi\text{-}OsMADS17}$ plants. Data are means ± s.d. (*n* = 20 plants), comparisons are made by two-tailed Student's *t*-test. Source data underlying Supplementary Fig. 8a, c-j are provided as a Source Data file.

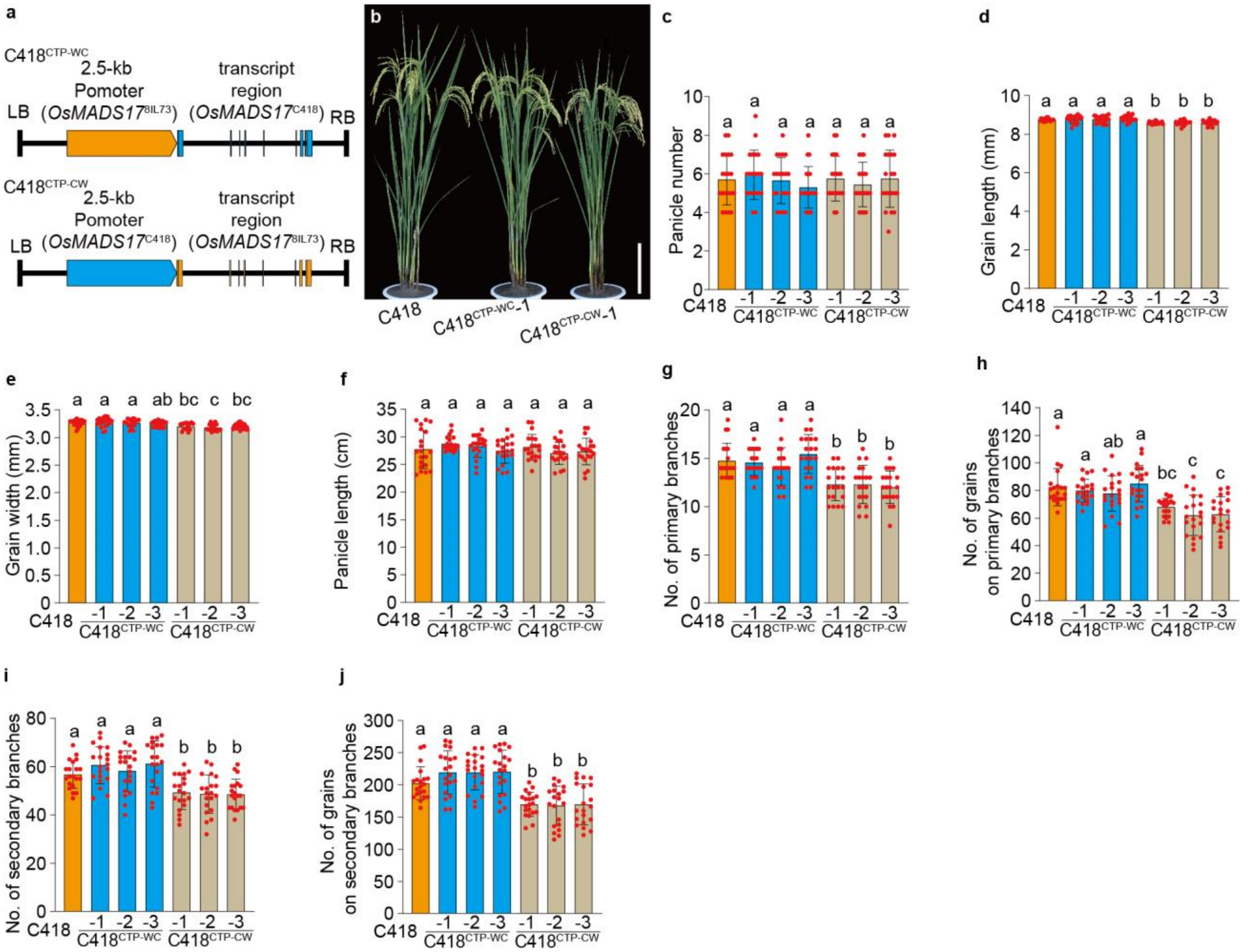

Supplementary Figure 9. Sequence variation in transcript region of *OsMADS17* was responsible for the phenotype difference between C418 and 8IL73. a Schematics of target fragments inserted into pCAMBIA1300 for generating complementary constructs. **b** The morphologies of C418, C418$^{CTP\text{-}WC}$, and C418$^{CTP\text{-}CW}$ plants. Scale bar, 25 cm. **c-j** Comparison of panicle number (**c**), grain length (**d**), grain width (**e**), panicle length (**f**), number of primary branches (**g**), grain number on primary branches (**h**), number of secondary branches (**i**), and grain number on secondary branches (**j**) among C418, C418$^{CTP\text{-}WC}$, and C418$^{CTP\text{-}CW}$ plants (n = 20 plants). Data are means ± s.d. The statistical significance was determined by one-way ANOVA with Tukey's multiple comparisons test, different letters represent significant differences (P < 0.05), and the exact P values were provided in Supplementary Data 1. Source data underlying Supplementary Fig. 9c-j are provided as a Source Data file.

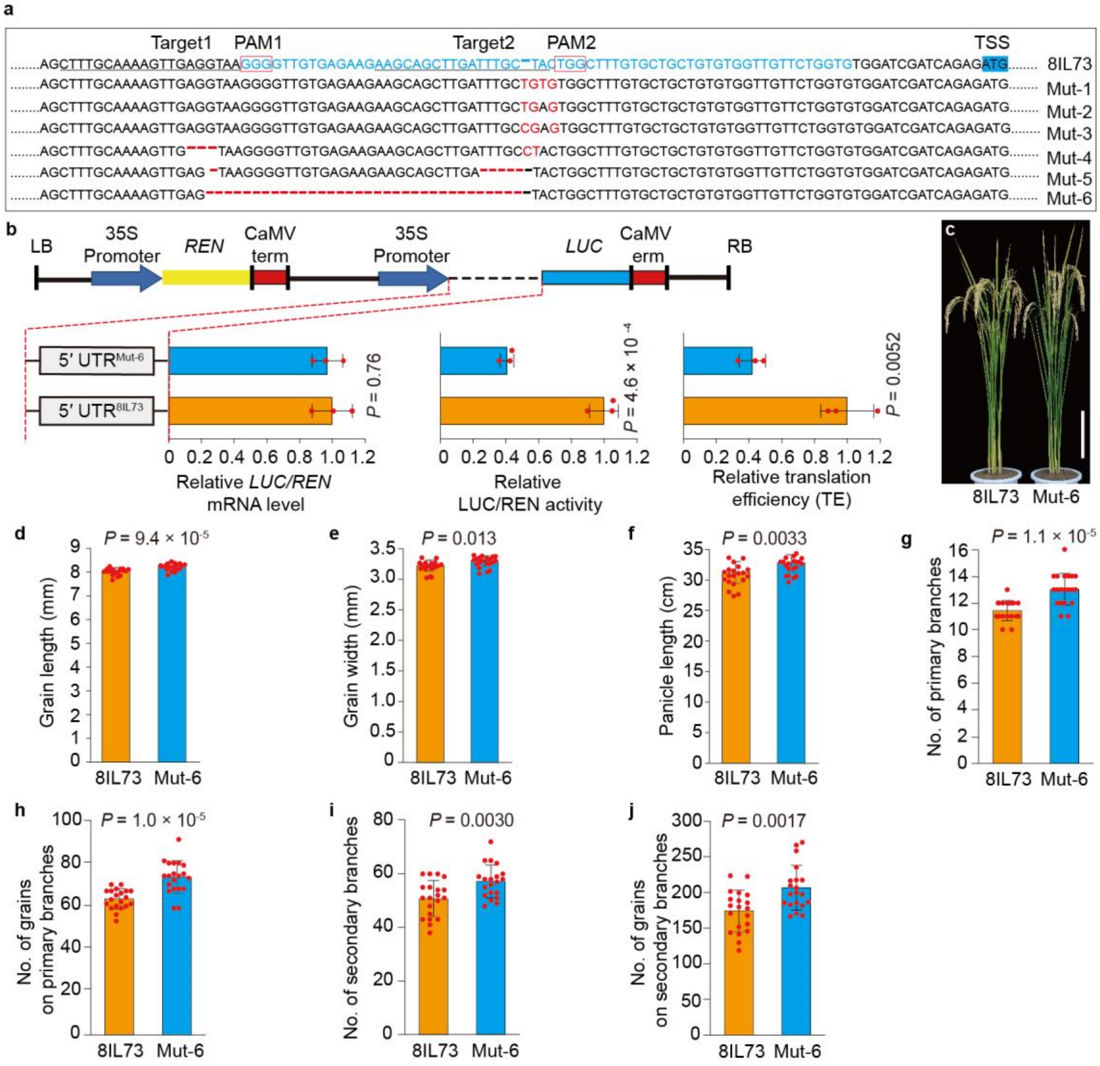

Supplementary Figure 10. A 34-bp deletion in the 5′ UTR of the *OsMADS17* gene in Mut-6 increased grain yield. a Different homozygous mutants obtained by CRISPR/Cas9 editing the 65-bp fragment. The blue letters indicate the 65-bp sequence, the red letters and lines indicate the mutated sites, the letters on the black line indicate the target sites, the letters in the red boxes indicate protospacer adjacent motif (PAM), and the letters in the blue box indicate the translation starting site (TTS). **b** A deletion of 34-bp in 5′ UTR of the *OsMADS17* gene in Mut-6 decreased translation efficiency (*n* = three replicates). **c** The morphologies of 8IL73 and Mut-6 plants. Scale bar, 25 cm. **d-j** Comparison of grain length (**d**), grain width (**e**), panicle length (**f**), number of primary branches (**g**), grain number on primary branches (**h**), number of secondary branches (**i**), and grain number on secondary branches (**j**) between 8IL73 and Mut-6 plants (*n* = 20 plants). Data are means ± s.d., comparisons are made by two-tailed Student's *t*-test. Source data underlying Supplementary Fig. 10b, d-j are provided as a Source Data file.

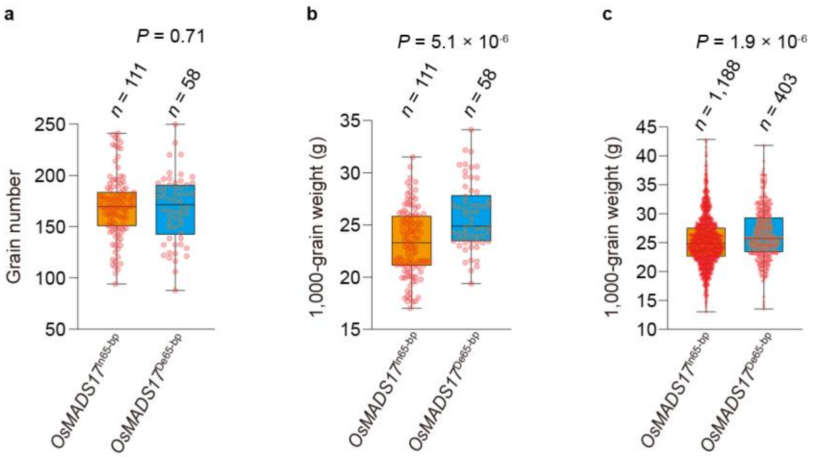

Supplementary Figure 11. The cultivars with the genotype *OsMADS17*$^{\text{De65-bp}}$ had heavier 1,000-grain weight than those with the genotype *OsMADS17*$^{\text{In65-bp}}$. a 169 cultivars were used for comparing the grain number between the cultivars with the genotype *OsMADS17*$^{\text{De65-bp}}$ and those with the genotype *OsMADS17*$^{\text{In65-bp}}$. **b**, **c** 169 cultivars (**b**) and 1,591cultivars from 3K RG (**c**) were used for comparing the 1,000-grain weight between the cultivars with the genotype *OsMADS17*$^{\text{De65-bp}}$ and those with the genotype *OsMADS17*$^{\text{In65-bp}}$. The box plots show all data points, two ends of the whiskers represent the minimum and the maximum value, and the middle, upper, and lower box lines represent the median and two quartiles of values in each group. *P*-values were calculated by two-tailed Student's *t*-tests. Source data are provided as a Source Data file.

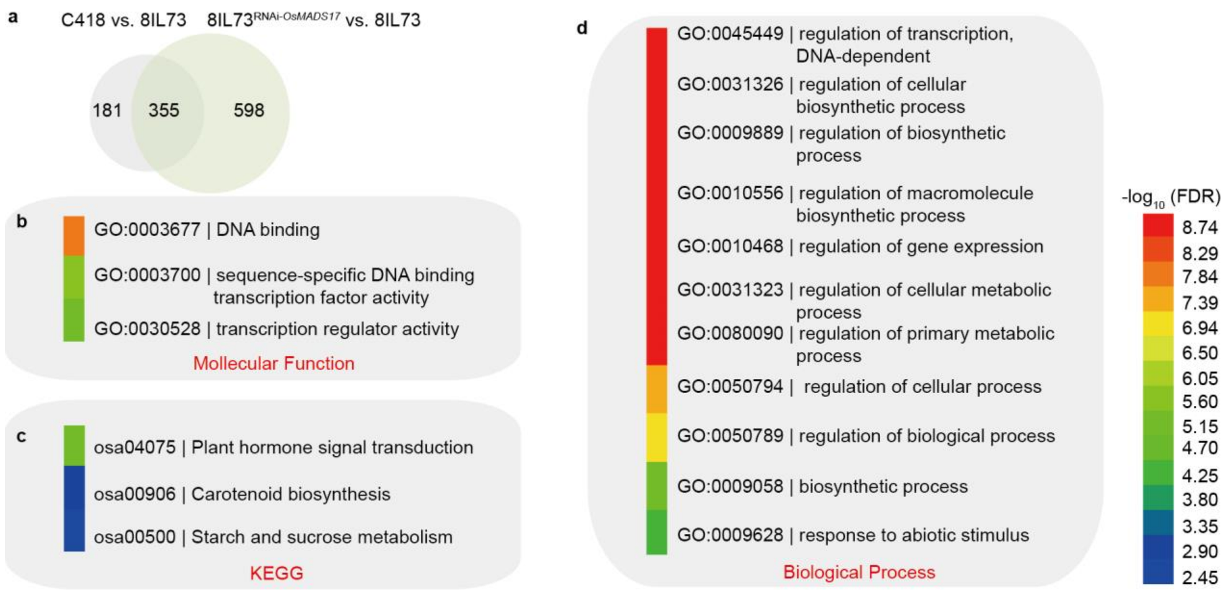

Supplementary Figure 12. *OsMADS17* participated in multiple regulatory pathways. a A total of 536 (C418 vs. 8IL73) and 953 (8IL73$^{\text{RNAi-}\mathit{OsMADS17}}$ vs. 8IL73) differentially expressed genes (DEGs) were identified (ratio≥2, *q*-value < 0.01), and 355 DEGs co-existed between the two groups. Young panicles (2 mm–4 mm) from C418, 8IL73, and transgenic plants 8IL73$^{\text{RNAi-}\mathit{OsMADS17}}$ were collected for RNA extraction. **b-d** GO and KEGG analysis of the 355 overlapping DEGs. Source data are provided as a Source Data file.

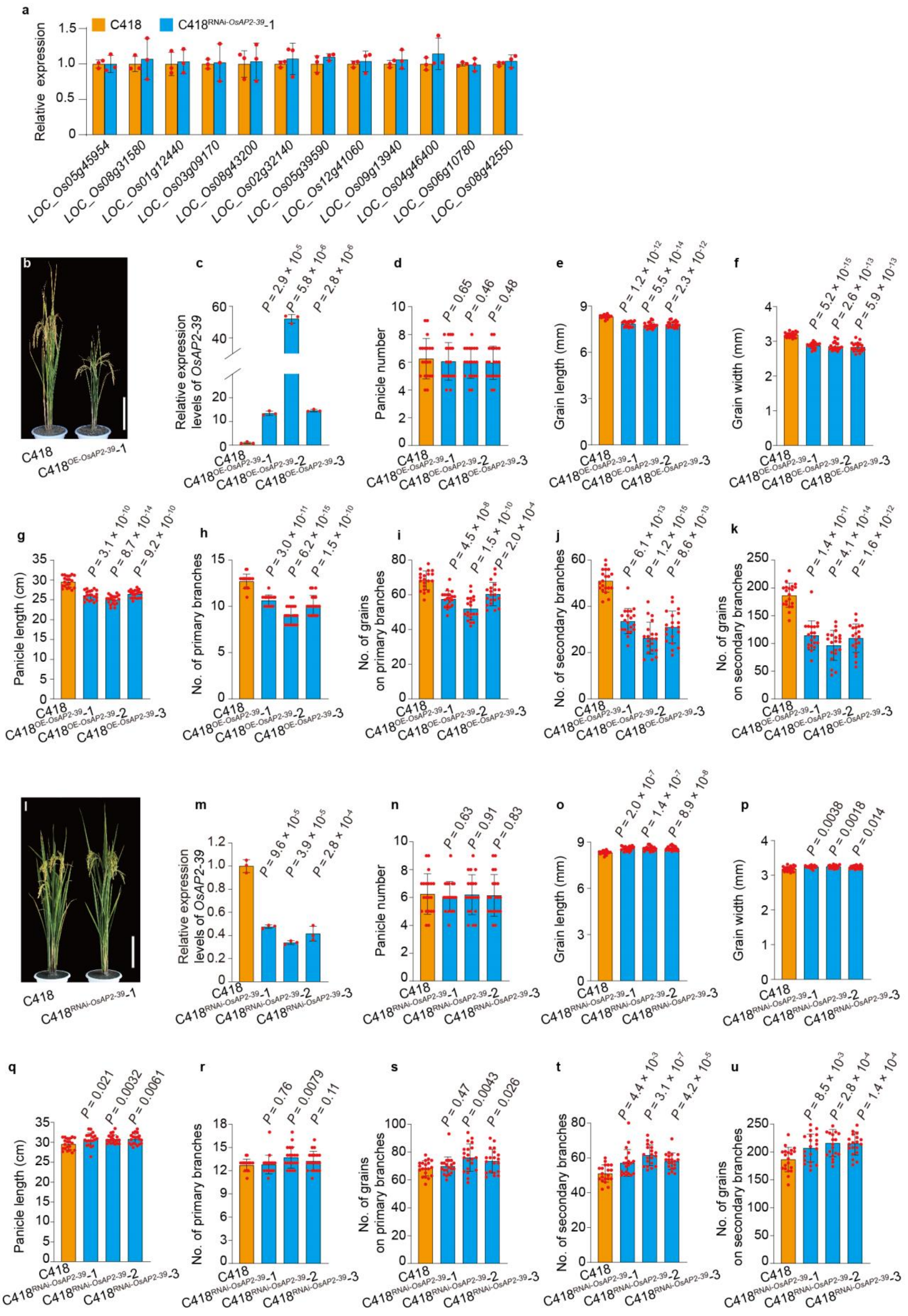
a
C418
C418RNAi-OsAP2-39-1
Relative expression
LOC_Os05g45954
LOC_Os08g31580
LOC_Os01g12440
LOC_Os03g09170
LOC_Os08g43200
LOC_Os02g32140
LOC_Os05g39590
LOC_Os12g41060
LOC_Os09g13940
LOC_Os04g46400
LOC_Os06g10780
LOC_Os08g42550
b
C418
C418OE-OsAP2-39-1
c
Relative expression levels of OsAP2-39
P = 2.9 × 10-5
P = 5.8 × 10-6
P = 2.8 × 10-6
d
Panicle number
P = 0.65
P = 0.46
P = 0.48
e
Grain length (mm)
P = 1.2 × 10-12
P = 5.5 × 10-14
P = 2.3 × 10-12
f
Grain width (mm)
P = 5.2 × 10-15
P = 2.6 × 10-13
P = 5.9 × 10-13
g
Panicle length (cm)
P = 3.1 × 10-10
P = 8.7 × 10-14
P = 9.2 × 10-10
h
No. of primary branches
P = 3.0 × 10-11
P = 6.2 × 10-15
P = 1.5 × 10-10
i
No. of grains on primary branches
P = 4.5 × 10-8
P = 1.5 × 10-10
P = 2.0 × 10-4
j
No. of secondary branches
P = 6.1 × 10-13
P = 1.2 × 10-15
P = 8.6 × 10-13
k
No. of grains on secondary branches
P = 1.4 × 10-11
P = 4.1 × 10-14
P = 1.6 × 10-12
C418
C418OE-OsAP2-39-1
C418OE-OsAP2-39-2
C418OE-OsAP2-39-3
l
C418
C418RNAi-OsAP2-39-1
m
Relative expression levels of OsAP2-39
P = 9.6 × 10-5
P = 3.9 × 10-5
P = 2.8 × 10-4
n
Panicle number
P = 0.63
P = 0.91
P = 0.83
o
Grain length (mm)
P = 2.0 × 10-7
P = 1.4 × 10-7
P = 8.9 × 10-8
p
Grain width (mm)
P = 0.0038
P = 0.0018
P = 0.014
q
Panicle length (cm)
P = 0.021
P = 0.0032
P = 0.0061
r
No. of primary branches
P = 0.76
P = 0.0079
P = 0.11
s
No. of grains on primary branches
P = 0.47
P = 0.0043
P = 0.026
t
No. of secondary branches
P = 4.4 × 10-3
P = 3.1 × 10-7
P = 4.2 × 10-5
u
No. of grains on secondary branches
P = 8.5 × 10-3
P = 2.8 × 10-4
P = 1.4 × 10-4
C418
C418RNAi-OsAP2-39-1
C418RNAi-OsAP2-39-2
C418RNAi-OsAP2-39-3

Supplementary Figure 13. *OsAP2-39* regulated rice grain yield negatively. a Identification of specificity for *OsAP2-39* specific RNA interference (RNAi) vector by testing the expression levels of 12 AP2 family genes in transgenic and the control plants (*n* = three replicates). **b** The morphologies of C418 and C418$^{\text{OE-}\textit{OsAP2-39}}$ plants. Scale bar, 25 cm. **c** Relative expression levels of *OsAP2-39* in C418 and C418$^{\text{OE-}\textit{OsAP2-39}}$ plants (*n* = three replicates). **d**-**k** Comparison of panicle number (**d**), grain length (**e**), grain width (**f**), panicle length (**g**), number of primary branches (**h**), grain number on primary branches (**i**), number of secondary branches (**j**), and grain number on secondary branches (**k**) between C418 and C418$^{\text{OE-}\textit{OsAP2-39}}$ plants (*n* = 20 plants). **l** The morphologies of C418 and C418$^{\text{RNAi-}\textit{OsAP2-39}}$ plants. Scale bar, 25 cm. **m** Relative expression levels of *OsAP2-39* in C418 and C418$^{\textit{RNAi-OsAP2-39}}$ plants (*n* = three replicates). **n-u** Same as (**d**) to (**k**) for C418 and C418$^{\text{RNAi-}\textit{OsAP2-39}}$ plants (*n* = 20 plants). Data are means ± s.d., comparisons are made by two-tailed Student’s *t*-test. Source data underlying Supplementary Fig. 13a, c-k, m-u are provided as a Source Data file.

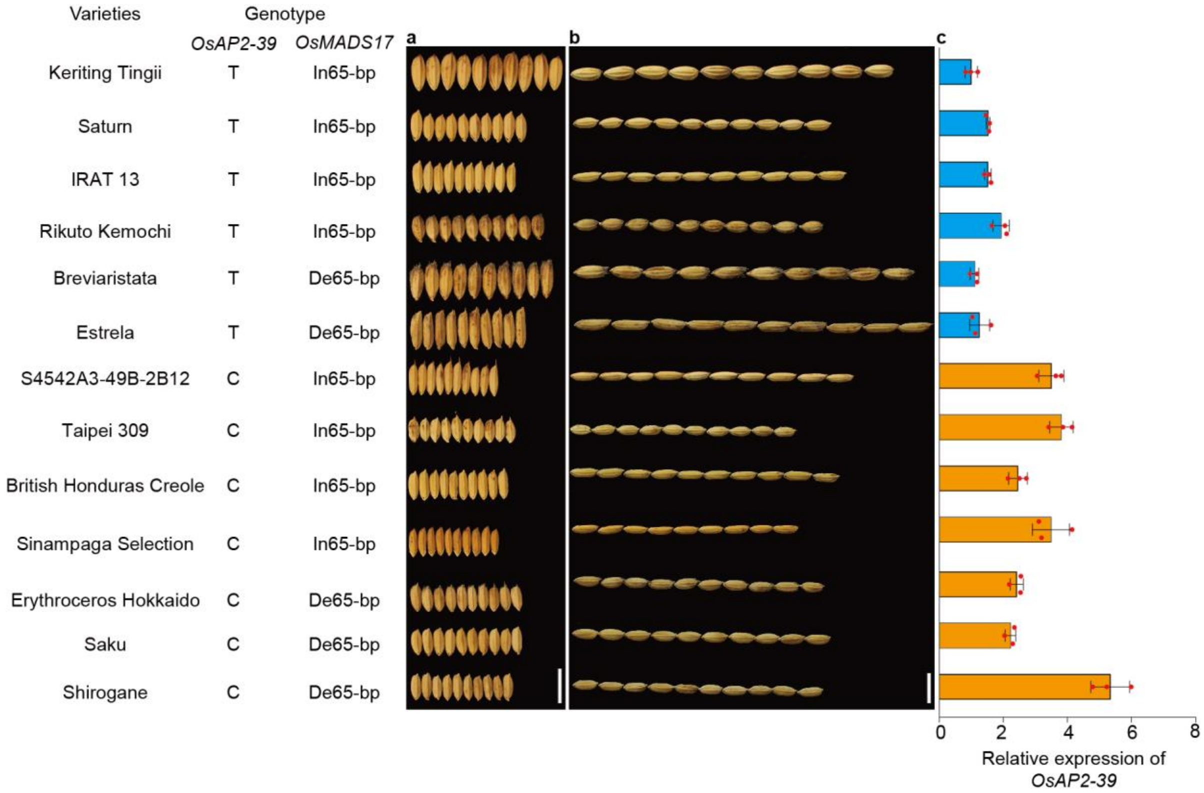

Supplementary Figure 14. Variation in the promoter region of *OsAP2-39* increased grain weight. a, **b** Grain width (**a**) and grain length (**a**) of cultivars with the genotype *OsAP2-39*$^{T/C}$ and *OsMADS17*$^{In65\text{-}bp/De65\text{-}bp}$. Scale bars, 1 cm. **c** Expression levels of *OsAP2-39* in cultivated rice accessions. Cultivars with *OsAP2-39*^{T} showed lower expression levels of *OsAP2-39* than those with *OsAP2-39*^{C} (*n* = three replicates). Data are means ± s.d. Source data are provided as a Source Data file.

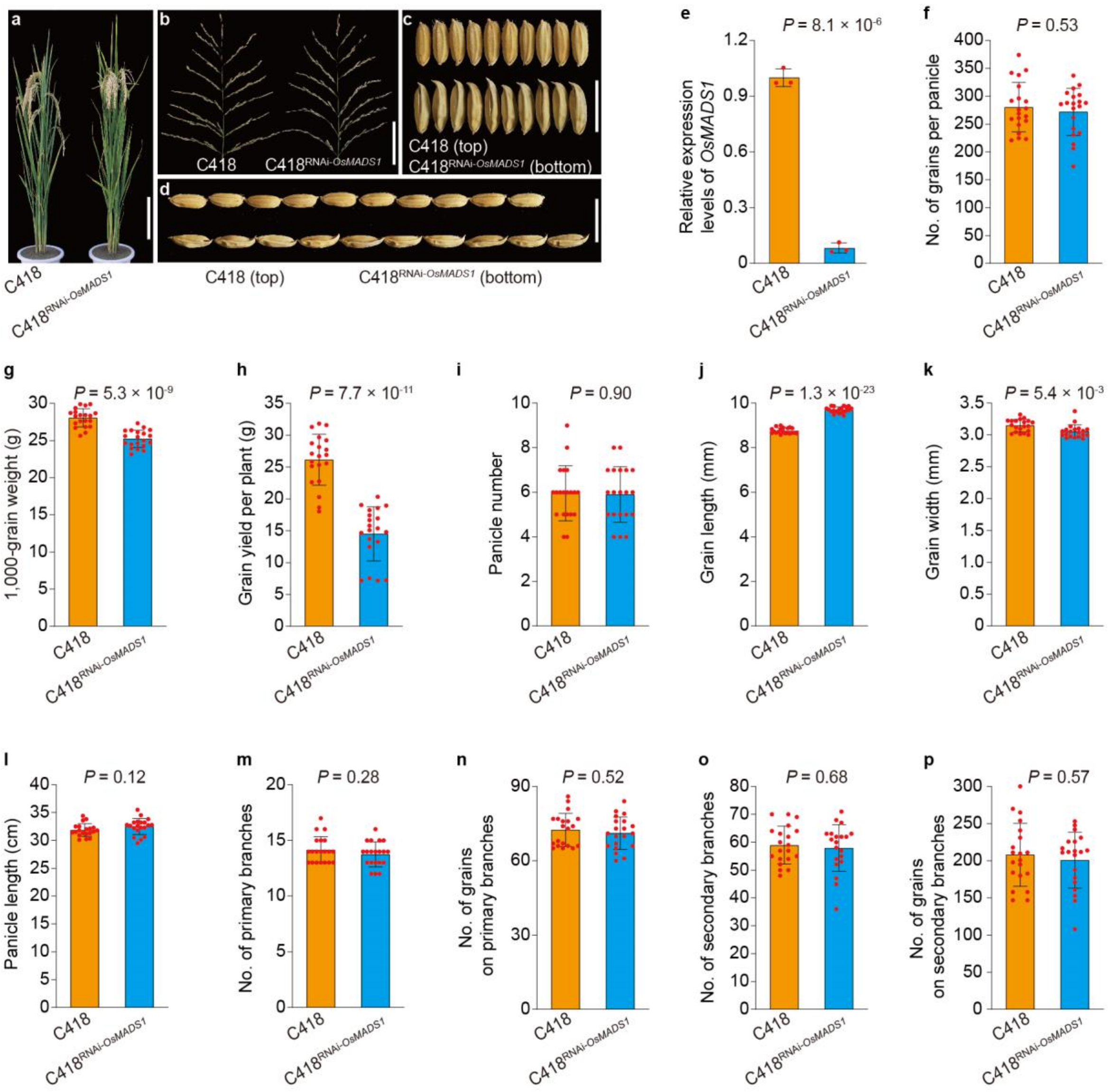

Supplementary Figure 15. Down-regulated expression levels of *OsMADS1* increased grain length. a-d The whole plant (**a**), main panicle (**b**), grain width (**c**), and grain length (**d**) of C418 and C418$^{\text{RNAi-}\textit{OsMADS1}}$ plants. Scale bars, 25 cm (**a**), 10 cm (**b**), and 1 cm (**c**, **d**). **e** Expression levels of *OsMADS1* in C418 and C418$^{\text{RNAi-}\textit{OsMADS1}}$ plants (*n* = three replicates). **f-p** Comparison of grain number (**f**), 1,000-grain weight (**g**), grain yield per plant (**h**), panicle number (**i**), grain length (**j**), grain width (**k**), panicle length (**l**), number of primary branches (**m**), grain number on primary branches (**n**), number of secondary branches (**o**), and grain number on secondary branches (**p**) between C418 and C418$^{\text{RNAi-}\textit{OsMADS1}}$ plants. Data are means ± s.d. (*n* = 20 plants), comparisons are made by two-tailed Student's *t*-test. Source data underlying Supplementary Fig. 15e-p are provided as a Source Data file.

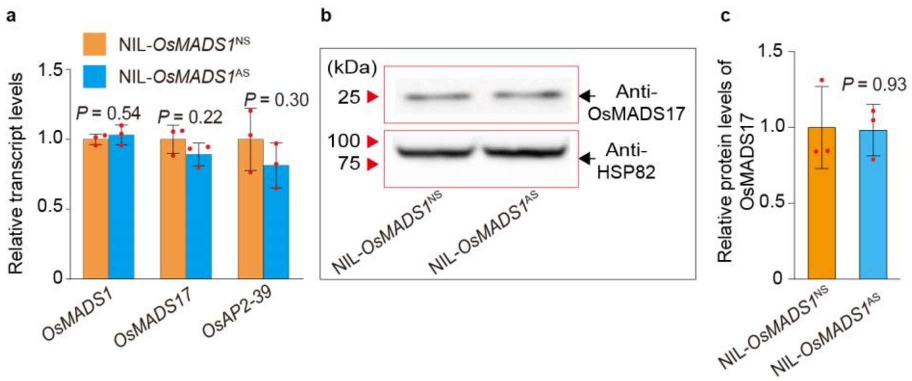

Supplementary Figure 16. The allelic variation of *OsMADS1*$^{NS/AS}$ did not affect the expression levels of *OsMADS17* and *OsAP2-39*. a The expression levels of *OsMADS1*, *OsMADS17*, and Os*AP2-39* were not significantly different between NIL-*OsMADS1*^{NS} and NIL-*OsMADS1*^{AS} plants. The NIL-*OsMADS1*^{NS} plants with the native splicing of *OsMADS1* which was same to that in *japonica* rice variety Nipponbare. The NIL-*OsMADS1*^{AS} plants with the natural variation of alternative splicing of *OsMADS1*. **b** Western blot analysis for OsMADS17 in NIL-*OsMADS1*^{NS} and NIL-*OsMADS1*^{AS} plants. **c** Comparison of relative expression levels of OsMADS17 between NIL-*OsMADS1*^{NS} and NIL-*OsMADS1*^{AS} plants. Data are means ± s.d. (n = three replicates), comparisons are made by two-tailed Student's t-test. Source data are provided as a Source Data file.

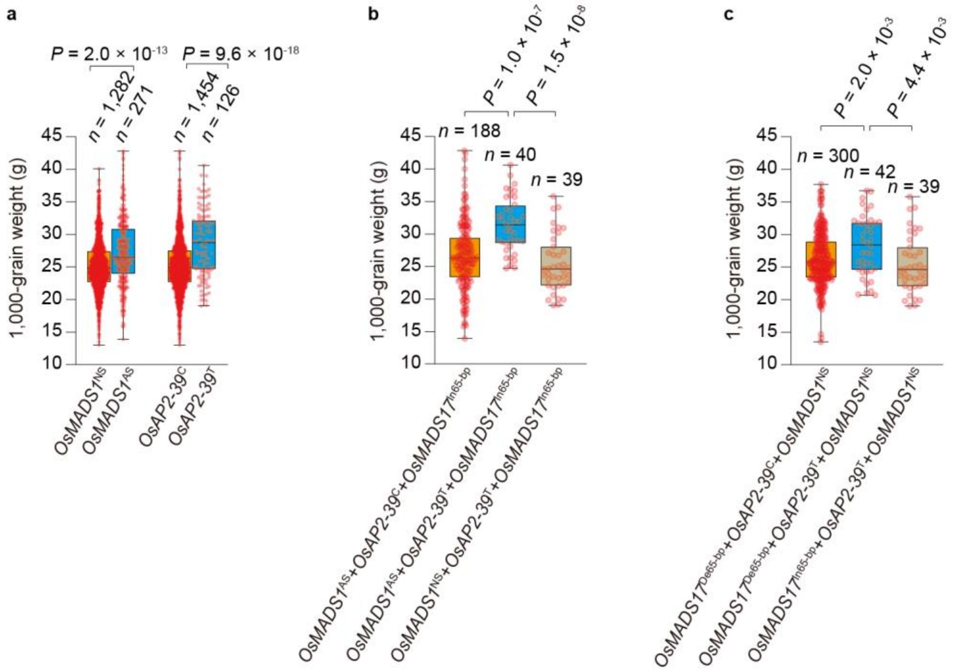

Supplementary Figure 17. *OsMADS1*, *OsMADS17*, and *OsAP2-39* co-regulate grain size. a Cultivars with the genotype *OsMADS1*^{AS} or *OsAP2-39*^{T} showed higher 1,000-grain weight compared with those with the genotype *OsMADS1*^{NS} or *OsAP2-39*^{C}, respectively. **b** Cultivars with the genotype *OsMADS1*^{AS}+*OsAP2-39*^{T} had heavier 1,000-grain weight than those with the genotype *OsMADS1*^{AS}+*OsAP2-39*^{C} or *OsMADS1*^{NS}+*OsAP2-39*^{T}. **c** Cultivars with the genotype *OsMADS17*$^{De65\text{-}bp}$+*OsAP2-39*^{T} displayed bigger individual grain size than those with the genotype *OsMADS17*$^{De65\text{-}bp}$+*OsAP2-39*^{C} or *OsMADS17*$^{In65\text{-}bp}$+*OsAP2-39*^{T}. The box plots show all data points, two ends of the whiskers represent the minimum and the maximum value, and the middle, upper, and lower box lines represent the median and two quartiles of values in each group. *P*-values were calculated by two-tailed Student's *t*-tests. Source data are provided as a Source Data file.

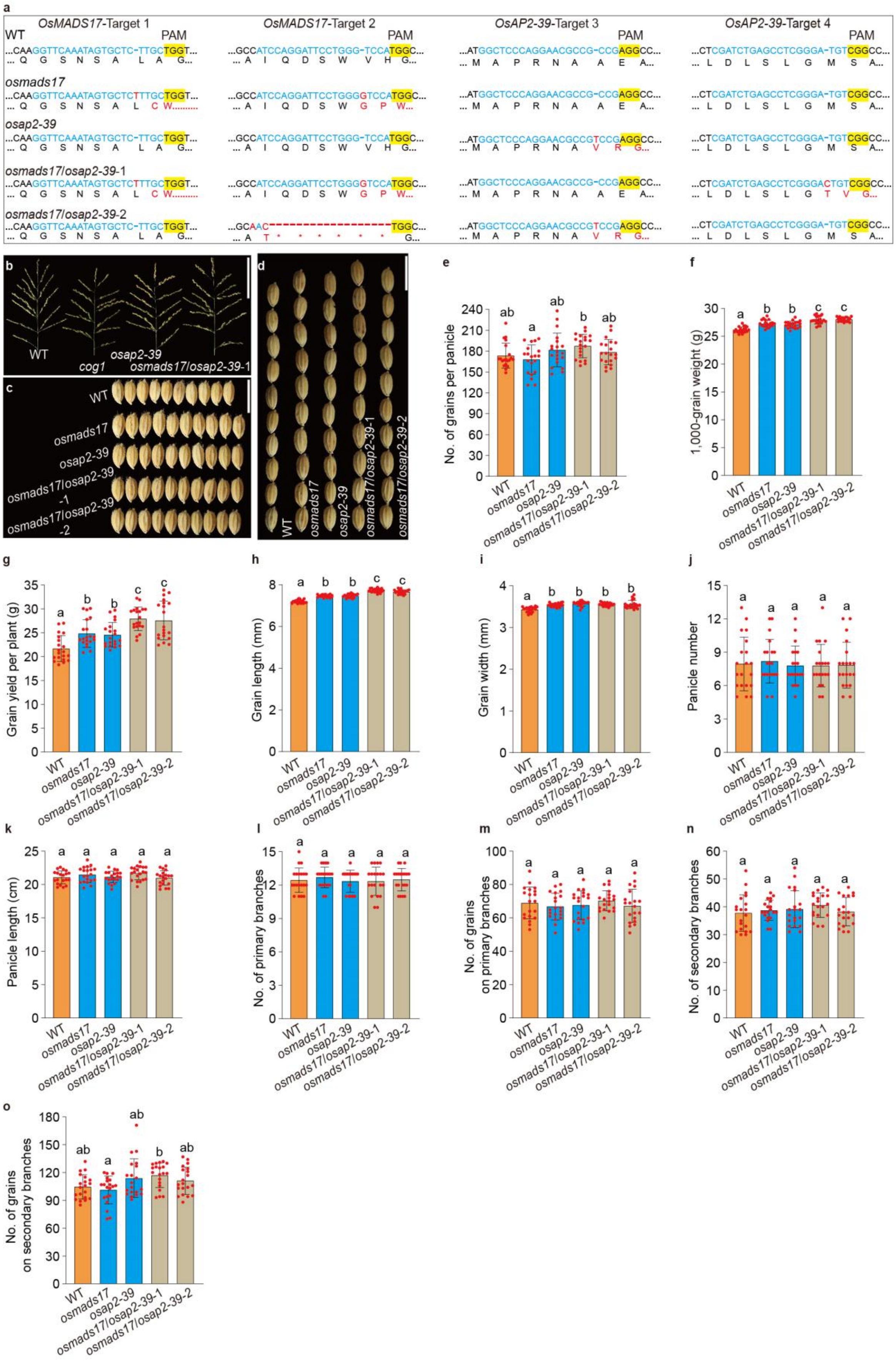
a
OsMADS17-Target 1
OsMADS17-Target 2
OsAP2-39-Target 3
OsAP2-39-Target 4
PAM
WT
osmads17
osap2-39
osmads17/osap2-39-1
osmads17/osap2-39-2
b
WT
cog1
osap2-39
osmads17/osap2-39-1
c
d
e
No. of grains per panicle
f
1,000-grain weight (g)
g
Grain yield per plant (g)
h
Grain length (mm)
i
Grain width (mm)
j
Panicle number
k
Panicle length (cm)
l
No. of primary branches
m
No. of grains on primary branches
n
No. of secondary branches
o
No. of grains on secondary branches

Supplementary Figure 18. Loss-of-function of *OsMADS17* and/or *OsAP2-39* increased grain yield. a Homozygous mutants obtained by CRISPR/Cas9 editing the coding region of the *OsMADS17* and *OsAP2-39* genes. The blue letters at the top and the black letters at the bottom indicate the targeted genomic sequences and amino acid sequences, respectively. The red letters, lines, and asterisks indicate the mutations, and the letters in the yellow boxes indicate protospacer adjacent motif (PAM). All mutants with single-base insertions at one or more sites resulted in a frameshift mutation. Two SNPs and a deletion of 18-bp in *osmads17*/*osap2-39*-2 (*OsMADS17*-Target2) led to a substitution of one amino acid and a deletion of six amino acids, respectively. **b-d** The main panicles (**b**), grain width (**c**), and grain length (**d**) of WT and mutants. Scale bars, 10 cm (**b**), and 1 cm (**c**, **d**). **e-o** Comparison of grain number (**e**), 1,000-grain weight (**f**), grain yield per plant (**g**), grain length (**h**), grain width (**i**), panicle number (**j**), panicle length (**k**), number of primary branches (**l**), grain number on primary branches (**m**), number of secondary branches (**n**), and grain number on secondary branches (**o**) among WT, *osmads17*, *osap2-39*, *osmads17*/*osap2-39*-1, and *osmads17*/*osap2-39*-2 plants. Data are means ± s.d. (n = 20 plants). The statistical significance was determined by one-way ANOVA with Tukey's multiple comparisons test, different letters represent significant differences ($P < 0.05$), and the exact P values were provided in Supplementary Data 4. Source data underlying Supplementary Fig. 18e-o are provided as a Source Data file.

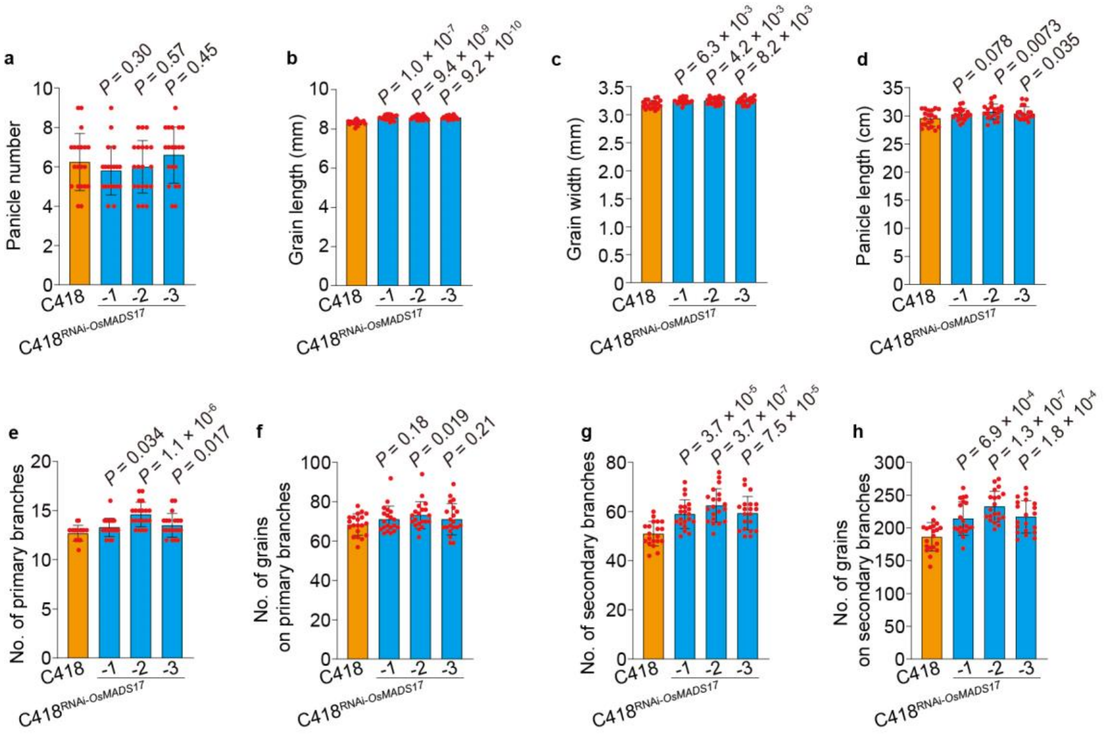

Supplementary Figure 19. Comparison of agronomic traits between C418 and C418$^{\textbf{RNAi-}\textit{\textbf{OsMADS17}}}$ plants. Comparison of panicle number (**a**), grain length (**b**), grain width (**c**), panicle length (**d**), number of primary branches (**e**), grain number on primary branches (**f**), number of secondary branches (**g**), and grain number on secondary branches (**h**) between C418 and C418$^{\text{RNAi-}\textit{OsMADS17}}$ plants (n = 20 plants). Data are means ± s.d., comparisons are made by two-tailed Student's t-test. Source data are provided as a Source Data file.

Supplementary Table 1. QTL analysis using 203 F_2 individuals.

Traits	Chr.	Markers	LOD	PV (%)	Add	*P*-value
No. of grains per main panicle	4	LT47	11.5	23	-29.89	0
1,000-grain weight (g)	4	LT47	6.28	14	-0.9	0
	6	LT6	1.98	5	0.5	0.011
	10	LT10-1	2.26	5	-0.54	0.005

QTL was detected by single-point analysis using the software Map Manager QTXb20. QTL: quantitative trait locus; LOD: likelihood of odd; PV: the phenotypic variance explained by the QTL; Add: additive effects. Statistical significance was analyzed by chi-square test. Source data are provided as a Source Data file.

Supplementary Table 2. DEGs related to regulating rice grain number and grain weight.

C418 vs. 8IL73						
Gene	Gene symbol	Value (C418)	Value (8IL73)	$\log_2$ (fold_change)	*p*_value	*q*_value
LOC_Os01g50410	*OsMKKK70*	2.14	4.67	1.13	5.0×10^{-5}	7.0×10^{-4}
LOC_Os04g33740	*GIF1*	161.05	66.04	-1.29	5.0×10^{-5}	7.0×10^{-4}
LOC_Os06g15620	*OsGSR1/GW6/OsGASR7*	305.47	661.25	1.11	5.0×10^{-5}	7.0×10^{-4}
LOC_Os08g06110	*OsCCA1*	156.05	23.32	-2.74	5.0×10^{-5}	7.0×10^{-4}
LOC_Os08g07010	*OsABCG18*	106.69	44.08	-1.28	5.0×10^{-5}	7.0×10^{-4}
LOC_Os08g37070	*OsCEP6.1*	23.72	48.20	1.02	1.5×10^{-4}	1.9×10^{-3}

Differentially expressed genes (DEGs) between C418 and 8IL73 were revealed by RNA-seq, which have been identified as regulators for rice grain number and grain weight. The differentially expressed genes were identified using a model based on the negative binomial distribution by the DESeq R package (1.18.0). *q*-value was the adjusted *p*-value by the Benjamini and Hochberg's approach for controlling the false discovery rate. Genes with *q*-value <0.01 were assigned as differentially expressed.
