# Supplementary material for: OsMADS17 simultaneously increases grain number and grain weight in rice
Source: Nat Commun. 2023 May 29;14:3098. doi: 10.1038/s41467-023-38726-9 (PMC10227085; doi:10.1038/s41467-023-38726-9)
Supplement: Supplementary file 3 — Description of Additional Supplementary files [file 41467_2023_38726_MOESM3_ESM.pdf]

Description of Additional Supplementary Files

File Name: Supplementary Data 1

Description: The statistical significance (*P* value) determined by one-way ANOVA with Tukey's multiple comparisons test for expression levels of *OsMADS17* and agronomic traits among C418, $C418^{CTP-WC}$, and $C418^{CTP-CW}$ plants.

File Name: Supplementary Data 2

Description: The statistical significance (*P* value) determined by one-way ANOVA with Tukey's multiple comparisons test for translation efficiency analysis of In/De65-bp.

File Name: Supplementary Data 3

Description: Wild rice accessions used in this study.

File Name: Supplementary Data 4

Description: The statistical significance (*P* value) determined by one-way ANOVA with Tukey's multiple comparisons test for agronomic traits among ZH17, *osmads17*, *osap2-39*, *osmads17*/*osap2-39*-1, and *osmads17*/*osap2-39*-2 plants.

File Name: Supplementary Data 5

Description: Primers used in this study.
