# Supplementary material for: OsMADS17 simultaneously increases grain number and grain weight in rice
Source: Nat Commun. 2023 May 29;14:3098. doi: 10.1038/s41467-023-38726-9 (PMC10227085; doi:10.1038/s41467-023-38726-9)
Supplement: Supplementary file 9 — Reporting Summary [file 41467_2023_38726_MOESM9_ESM.pdf]

nature portfolio

Corresponding author(s): Chuanqing Sun, Hongying Sun

Last updated by author(s): May 10, 2023

Reporting Summary

Nature Portfolio wishes to improve the reproducibility of the work that we publish. This form provides structure for consistency and transparency in reporting. For further information on Nature Portfolio policies, see our Editorial Policies and the Editorial Policy Checklist.

Statistics

For all statistical analyses, confirm that the following items are present in the figure legend, table legend, main text, or Methods section.

n/a	Confirmed	
☐	☒	The exact sample size (*n*) for each experimental group/condition, given as a discrete number and unit of measurement
☐	☒	A statement on whether measurements were taken from distinct samples or whether the same sample was measured repeatedly
☐	☒	The statistical test(s) used AND whether they are one- or two-sided<br>*Only common tests should be described solely by name; describe more complex techniques in the Methods section.*
☒	☐	A description of all covariates tested
☐	☒	A description of any assumptions or corrections, such as tests of normality and adjustment for multiple comparisons
☐	☒	A full description of the statistical parameters including central tendency (e.g. means) or other basic estimates (e.g. regression coefficient) AND variation (e.g. standard deviation) or associated estimates of uncertainty (e.g. confidence intervals)
☐	☒	For null hypothesis testing, the test statistic (e.g. *F*, *t*, *r*) with confidence intervals, effect sizes, degrees of freedom and *P* value noted<br>*Give P values as exact values whenever suitable.*
☒	☐	For Bayesian analysis, information on the choice of priors and Markov chain Monte Carlo settings
☒	☐	For hierarchical and complex designs, identification of the appropriate level for tests and full reporting of outcomes
☒	☐	Estimates of effect sizes (e.g. Cohen's *d*, Pearson's *r*), indicating how they were calculated

Our web collection on statistics for biologists contains articles on many of the points above.

Software and code

Policy information about availability of computer code

Data collection	CFX96 Real-Time System (Bio-Rad) or StepOnePlus (Applied Biosystems) (RT-qPCR data); Illumina HiSeq(TM)2000 (RNA-sequencing); Model SC-G, Wanshen (data for 1,000-grain weight, grain length, and grain width); Image J program (Image J 1.48, https://imagej.net/ij/) (Measuring western blot bands). All data were measured as described in the manuscript without any special software.
Data analysis	RNA-seq data were processed by HTSeq v0.9.1 for gene expression level analysis and DESeq R package (1.18.0) for differentially expressed genes analysis. The functional category analysis of the DEGs were performed using agriGO and KEGG (Yi et al., Nucleic Acids Res. 2013, 41: W98-W103; Tian et al., Nucleic Acids Res. 2017, 45: W122-W129). QTL analysis were performed by the Map Manager QTXb20. Association analysis was performed with Fisher’s exact test, and statistical analysis are made by two-tailed Student’s t-test or one-way ANOVA with Tukey’s multiple comparisons test (P<0.05) using SPSS version 17 (SPSS Inc., Chicago, IL, USA). The geographical distribution of cultivated rice in Asia areas was analyzed by map package (R v4.1.1).

For manuscripts utilizing custom algorithms or software that are central to the research but not yet described in published literature, software must be made available to editors and reviewers. We strongly encourage code deposition in a community repository (e.g. GitHub). See the Nature Portfolio guidelines for submitting code & software for further information.

March 2021

Data

Policy information about availability of data

All manuscripts must include a data availability statement. This statement should provide the following information, where applicable:

- Accession codes, unique identifiers, or web links for publicly available datasets
- A description of any restrictions on data availability
- For clinical datasets or third party data, please ensure that the statement adheres to our policy

Data of genotype, phenotype (1,000-grain weight), subgroup classification, and origin for rice accessions are available on RFGB v2.0 database (https://www.rmbreeding.cn/). Information on gene sequence and annotation are available on Rice Genome Annotation Project (http://rice.uga.edu/). The authors declare that the data supporting the findings of this study are available within the paper and the Supplementary Information. Source data are provided with this paper.

Human research participants

Policy information about studies involving human research participants and Sex and Gender in Research.

Reporting on sex and gender	N/A
Population characteristics	N/A
Recruitment	N/A
Ethics oversight	N/A

Note that full information on the approval of the study protocol must also be provided in the manuscript.

Field-specific reporting

Please select the one below that is the best fit for your research. If you are not sure, read the appropriate sections before making your selection.

☒ Life sciences ☐ Behavioural & social sciences ☐ Ecological, evolutionary & environmental sciences

For a reference copy of the document with all sections, see nature.com/documents/nr-reporting-summary-flat.pdf

Life sciences study design

All studies must disclose on these points even when the disclosure is negative.

Sample size	No statistic methods were used to predetermine sample size. Required experimental sample size were estimated based on our past experience performing similar experiments including field test (Huang et al., Plant J. 2018, 96: 716-733; Huo et al., Nat. Commun. 2017, 8: 1497).
Data exclusions	No data was excluded from the analysis.
Replication	All experiments were successfully repeated at least three times.
Randomization	Measurements and samplings were performed by randomly selecting plants grown under the exact same conditions. All samples were allocated randomly into experimental groups.
Blinding	We did not apply blinding as it was not applicable for the nature of the experimental setup.

Reporting for specific materials, systems and methods

We require information from authors about some types of materials, experimental systems and methods used in many studies. Here, indicate whether each material, system or method listed is relevant to your study. If you are not sure if a list item applies to your research, read the appropriate section before selecting a response.

Materials & experimental systems

n/a	Involved in the study
☐	☒ Antibodies
☒	☐ Eukaryotic cell lines
☒	☐ Palaeontology and archaeology
☒	☐ Animals and other organisms
☒	☐ Clinical data
☒	☐ Dual use research of concern

Methods

n/a	Involved in the study
☒	☐ ChIP-seq
☒	☐ Flow cytometry
☒	☐ MRI-based neuroimaging

Antibodies

Antibodies used	Anti-OsMADS17 (generated in rabbit using a specific fragment consisting of OsMADS17 amino acids (C-NKINRQVTFSKRRN) by AbMART, 1:2,000 dilution) was used for western blot analysis; Anti-MBP (Cat# AbM59007-3-PU, Beijing Protein Innovation (BPI), 1:1,000 dilution) was used for detecting the fused protein MBP-OsMADS17 by western blot analysis; Anti-HSP82 (Cat# AbM51099-31-PU, Beijing Protein Innovation (BPI), 1:5,000 dilution) was used for western blot as loading control; Antibody to Firefly Luciferase (Anti-LUC) (Cat# T55401, AbMART, 1:1,000 dilution) and Renilla Luciferase (Anti-REN) (Cat# T55403, AbMART, 1:1,000 dilution) were used for western blot analysis.
Validation	Anti-OsMADS17 was validated in vitro experiment using recombinant protein of MBP-OsMADS17 as described in Figure 3h. The Anti-MBP, Application: WB, Elisa, more details in http://www.proteomics.org.cn/product/189.html. The Anti-HSP82, Species Reactivity: Wheat, Corn, Cotton, Arabidopsis, Rice, Application: WB, Elisa, more details in http://www.proteomics.org.cn/product/202.html. The Anti-LUC, Species Reactivity: Firefly, Application: WB, ICC/I, Specificity: detects endogenous levels of total Firefly Luciferase, more details in http://www.ab-mart.com.cn/page.aspx?node=%2077%20&id=%201670. The Anti-REN, Species Reactivity: Renilla Luciferase, Application: WB, ICC/I, Specificity: detects endogenous levels of total Renilla Luciferase, more details in http://www.ab-mart.com.cn/page.aspx?node=%2077%20&id=%201672. Antibodies are commercially available from the manufactures.
